# Health-related quality of life and cost-of-illness in young people seeking peer support at @ease: A Dutch burden of disease study

**DOI:** 10.1371/journal.pone.0352652

**Published:** 2026-07-06

**Authors:** Anouk Boonstra, Ghislaine A. P. G. van Mastrigt, Silvia M. A. A. Evers, Sander Osstyn, Remco F. P. de Winter, Nynke Boonstra, @ease consortium, Therese A. M. J. van Amelsvoort, Sophie M. J. Leijdesdorff

**Affiliations:** 1 Mental Health and Neuroscience (MHeNs) Research Institute, Department of Psychiatry and Neuropsychology, Maastricht University, Maastricht, the Netherlands; 2 Care and Public Health Research Institute (CAPHRI), Department of Health Services Research, Maastricht University, Maastricht, the Netherlands; 3 Alzheimer Centre Limburg, Faculty of Health Medicine and Life Sciences, Mental Health and Neuroscience Research Institute, Department of Psychiatry and Neuropsychology, Maastricht University, Maastricht, the Netherlands; 4 Mental Health Institute Rivierduinen, Leiden, the Netherlands; 5 Section Clinical Psychology, Vrije Universiteit Amsterdam, Amsterdam, the Netherlands; 6 Department of Healthcare, NHL Stenden University of Applied Sciences, Leeuwarden, the Netherlands; 7 KieN VIP Metal Health Care Services, Leeuwarden, the Netherlands; 8 Department of Psychiatry, UMC Utrecht Brain Center, Utrecht, the Netherlands; Grand Canyon University, UNITED STATES OF AMERICA

## Abstract

**Introduction:**

The burden of mental health problems remains largely unexplored among vulnerable young people, especially those seeking peer support. Accessing peer support is often a first form of help-seeking, allowing early identification of signs of distress. The lost (mental) health and expenses of these young people upon presenting for peer support can be revealed through monitoring of health-related quality of life (HRQoL) and costs of mental healthcare and productivity loss, examined in this study among young people visiting the @ease peer-to-peer walk-in centres in the Netherlands.

**Methods:**

From @ease’s inception in January 2018 to mid-2024, a total of 940 answered questionnaires gathered through consecutive sampling contained minimally one required item. This bottom-up prevalence-based study focused on youth aged 12–30 who sought peer counselling at @ease. Burden of disease was estimated by: (1) HRQoL (EQ-5D-5L), and (2) Cost-of-illness through school absenteeism and mental healthcare use. Multiple imputation was used before conducting regression analyses, followed by non-parametric bootstrapping. This study expands upon an earlier publication that analysed data up to May 2019.

**Results:**

HRQoL was impaired (*M* = 0.64, *SD* = 0.24) and significantly lower if living alone or having parents with mental health problems, and higher if having a higher level of social and occupational functioning. In the three months before presenting, 35.2% of young people had been absent from school (3 days on average, costing €358 per individual) and 33.4% had visited mental healthcare (2 visits on average, costing €234 per individual). Total cost-of-illness was €1,501,743 annually, and €2,318 per individual. Mental healthcare costs were higher for those born in the Netherlands and without occupation, and school absenteeism costs were higher outside the COVID-19 pandemic and if not born in the Netherlands.

**Conclusions:**

Found impairments and costs underscore the importance of investing in early-stage low-threshold services where substantial burden is already detectable, and of strengthening their capacity and links to stepped-care pathways to ensure timely support. Initiatives that help improve functioning and aid with challenging contexts may be advantageous in lowering the burden. Prospective (cost-)effectiveness studies are needed.

## Introduction

From an early age, mental ill-health contributes substantially to disability and morbidity: mental disorders begin before the age of 25 in 62.5% of cases, with the highest incidence around age 14.5 [1], and the leading causes of disability-adjusted life-years among young people include self-harm, depressive disorders and anxiety disorders [2]. To prevent (further) impairment and higher societal costs, it is crucial to invest in, and improve upon, early detection and intervention. This need is also high from a societal perspective, given that mental disorders pose major challenges for health systems and further impact the economy through productivity losses [3].

The impact of ill-health, termed ‘burden of disease’, is typically studied by assessing health-related quality of life (HRQoL) and cost-of-illness (COI). These universal measures support priority-setting and resource allocation within health systems. Impairments in HRQoL were found among young people with existing mental disorders [4–10] and constitute a potential risk factor for youth suicide [11]. However, much less is known about COI among young people, while the quantification of economic burden provides essential evidence about the scale of the financial impact, as well as for planning, prioritising, and resourcing youth mental health services and the design of responsive early‑intervention services.

A recent review of economic studies on loneliness and social isolation reported finding no research conducted among young adults, while the prevalence is higher than in adult groups which are studied more frequently [12]. While Meisters et al. [13] found that loneliness was associated with higher mental healthcare costs among the younger population, they included up to age 40. For mental healthcare use in general, Dijkstra et al. [14] found that costs were higher among younger adults aged 18–34 than older adults aged 35–65 in the Netherlands. However, much remains unknown about the burden of disease of young people with mental vulnerabilities in the age range of 12–25 years, both in terms of HRQoL and COI.

National research showed that 55.2% of young people in the Netherlands have experienced mental ill-health in their lifetime [15], but this figure included mood-, anxiety- and substance disorders and attention deficit hyperactivity disorder (ADHD). In addition, little is known about subthreshold cases, which must be incorporated in the calculation of public health burden [16]. As such, the burden for young people aged 12–25 with mental vulnerabilities was studied by Leijdesdorff et al. [17] among youth who sought peer support at the @ease walk-in centres in the Netherlands. This research provided insights into a vulnerable group that might otherwise have remained undetected as many had not received support beforehand, whilst over 90% had clinical levels of psychological distress [18,19]. Leijdesdorff et al. [17] found that the average HRQoL among these young peer-support seekers was comparable to that of people with a full-threshold depression and lower than in the general population [17]. The financial burden was evident as well: costs of mental healthcare use and productivity loss in terms of school absenteeism amounted to €32,809 over the past three months.

Given the lack of further studies in this area, the burden among at-risk young people remains largely undetected and indications for prevention needs remain undiscovered. As the sample size and time frame of the previous study by Leijdesdorff et al. [17] were restricted to the 80 participants who answered at least one question regarding a main outcome in the first 1.5 years, a larger study with extension of sample size was planned [20] and performed in the present study. The objective was to assess HRQoL and COI among young people seeking peer support at @ease and to explore potential risk groups, with COI being assessed from a limited societal perspective that entailed school absenteeism costs and mental healthcare costs.

## Methods

### Study design and setting

This cross-sectional prevalence-based study with a bottom-up costing approach, planned in a protocol [20], was performed across @ease centres in 11 cities throughout the Netherlands (Amsterdam [4 centres], Eindhoven, Groningen, Haarlem, Heerlen [2 centres], Leeuwarden, Leiden, Maastricht, Roermond, Rotterdam, Zwolle). @ease consists of accessible youth-tailored mental health centres, based on headspace Australia [21], but adjusted for exclusively offering walk-in peer support (www.ease.nl). Young people aged 12–25 years could walk into an @ease location freely and anonymously for a counselling session with two trained and professionally supervised young adult peers. The @ease working method is detailed elsewhere [17–20]. The research sample reflects those who attended this peer support service, which may differ from other help‑seeking or non‑help‑seeking youth. Moreover, not all eligible visitors may have completed the survey as participation was voluntary.

The COI-part was reported using the consensus-based checklist for the critical appraisal of COI studies [22] (See S5 File). In this study, COI was assessed from a limited societal perspective, incorporating direct costs of school absenteeism and mental healthcare use. While productivity loss is often measured as work absenteeism, school absenteeism was enquired instead, as most @ease visitors are in school or studying, and the questionnaire had to be as brief as possible. Furthermore, mental healthcare costs are a more direct reflection of the burden of disease for this population with mental health (and related) problems. The epidemiological approach was prevalence-based and the costing approach was bottom-up, as it started from individual-level data collected by means of first-visit questionnaires over 7 ½ years, with resulting estimations of the cost of 1 year for consistency and comparison with the previous study [17].

### Participants

Included were young people aged 12–30 years; whilst @ease is largely intended for 12–25-year-olds, age was not asked at the start of the conversation to maintain anonymity, leading to the inclusion of some young people marginally above the age of 25 years. Questionnaire data were included if @ease visitors agreed to participate, filled out at least one outcome measure, and if their visit was within the inclusion period of January 2018 to June 2024.

### Ethical considerations

At the end of every counselling conversation, the peer counsellors provided the young person with a routine questionnaire on a tablet device and asked for consent, clarifying first that participation is completely voluntary and that data are processed anonymously for research purposes. Data were automatically recoded to maintain anonymity and saved on a secure server of Maastricht University, accessible strictly only to this project’s researchers who kept full confidentiality and to whom no identifying variables were available. The dataset for the use of the present study was accessed on July 7^th^, 2024. The Medical Ethical Committee of Maastricht University has approved this research conducted at @ease and its procedure for being non-subjected to the Medical Research Involving Human Subjects Act (non-WMO) (METC number 2017−0046).

### Materials

The full questionnaire has been described in a protocol [20]. The main outcome of HRQoL was measured using the EQ-5D-5L which is an EuroQol health state questionnaire that is concise and widely used [23]. The five dimensions (5D) are *Mobility, Self-care, Usual Activities, Pain/discomfort and Anxiety/depression,* and each dimension has five answer levels (5L) ranging from *no problems (1)* to *not able to/extreme (5),* all phrased in sentences*.* The resulting health state (i.e., a sequence of the answers provided by the participant) is then converted into a total value (i.e., a utility). This utility specifies HRQoL on a universally comparable scale where 0 means “death”, 1 “full health”, and negative scores indicate a state worse than death. In this study, utilities were calculated using the Dutch population values [24].

Young people were asked: (1) whether and how many days they had been absent from school; and (2) whether and how often (i.e., number of sessions/appointments) they visited a healthcare professional for mental health problems or addiction problems during the last three months. The questionnaire only stored the number of days for participants who answered ‘yes’. Hence, a zero was inserted for zero days of school absenteeism or received care for everyone who answered ‘no’.

The peer counsellors evaluated the young person’s social and occupational functioning (SOFAS) which is a single score between 0 and 100, where 100 indicates superior functioning and every 5^th^ score can be chosen (e.g., ’65’ or ‘70’, not ’61’). The SOFAS was incorporated as possible predictor of HRQoL in the original and present study, not as a main outcome.

### Cost calculations

All costs, calculated in euros (€), and the step-by-step process are detailed in S1 File, including inflation correction and resulting unit prices. First, the annual societal cost of school absenteeism was derived from Statistics Netherlands [25]. This cost was divided by the number of students reported by Statistics Netherlands [26], separately for each educational level, and converted to costs per day. The cost of one day of absence was multiplied by each @ease visitor’s number of self-reported absent days in the three months before visiting @ease and averaged across all visitors.

Annual costs of mental healthcare use were calculated by dividing the costs within the Dutch costing manual [27] by the number of consultations within the Netherlands Mental Health Survey and Incidence Study-3 (NEMESIS-3) [28], once for general practitioners (GPs), once for psychologists, and then averaged. This weighted average was calculated because the specific kind of consulted healthcare professional was not enquired in the questionnaire. Thereafter, the cost of one consultation of one person was multiplied by the self-reported number of consultations that each @ease visitor had had in the three months before visiting @ease and then averaged.

Next, costs were calculated per calendar year, and school absenteeism and care costs were summed. Calculations were identical to Leijdesdorff et al. [17], but with a more recent costing manual [29] and incidence survey [28], and more recent data from Statistics Netherlands [25,26]. Details of the calculations have been described in the protocol [20].

### Analyses

Analyses were conducted using IBM SPSS Statistics 29.0.2.0, except for bootstrapping, which was done in Excel (Microsoft 365 Apps for Enterprise). In preparation of analysing, categorial variables were dummy-coded. Gender was recoded into three separate binary variables (yes/no). Living situation was recoded into 1 = living alone and 0 = other living situations. Education was recoded into theoretical (encompassing senior general secondary education (HAVO), pre-university education (VWO), university (WO) and higher professional education (HBO)) or vocational (encompassing pre-vocational secondary education (VMBO) and secondary vocational education (MBO)), where one was coded 1 and the other 0, depending on the hypothesized direction also specified in the output tables.

First, descriptive analyses were conducted. Next, correlation coefficients were calculated within and between all potential predictors and outcomes using pairwise complete observations (>.07 classified as strong, .04-.07 as moderate, and <.04 as weak). Following this, potential predictors were examined using multiple regression analyses by the Enter method to uncover potential risk groups for a higher burden of disease, with Bonferroni correction for multiple testing resulting in a significance threshold of *a* = .013. For each regression model, we planned to report the regression coefficients (*B*), their standard errors (*SE*), *t*-values, and associated *p*-values. Po*t*ential predictors of utilities and costs were specified earlier [20]. For mental healthcare costs, three additional potential predictors were added as found in literature. More specifically, mental health service use was high for children of parents with mental health problems [30], as well as during the COVID-19 pandemic [31], and having no occupation was found to be closely linked to poor mental health [32]. Before analysing, missing data (costs, utilities and predictors) were imputed 100 times using multiple imputation (MI) with fully conditional specification (FCS), a powerful, efficient, flexible and statistically valid approach for imputing both continuous and discrete variables, which increases precision and reduces biases compared to a complete case analysis [33,34]. For each analysis, the 100 results were pooled into a single outcome. For comparison, a complete case analysis was also applied as in the previous study to check results of the imputed data with those of the raw data and detect potential differences.

Given the non-normal distribution of the cost data, linear regression may be less statistically sound given the sample size. Hence, non-parametric bootstrapping was applied on the cost data per subgroup combination. Subgroups were the same as the hypothesized predictors [20] and as specified above.

## Results

Between January 2018 and June 2024, 940 young people completed minimally one outcome measure in the questionnaires at their first or sole visit to @ease. The majority were female (61.6%) and lived with parents (37.0%), peers (26.0%) or alone (25.8%). The average age was 20.3 years (*SD* = 3.5). Most young people were following an education (77.3%) and most frequently at tertiary level (73.5%), see Table 1. Furthermore, while 289 (36.0%) of young people reported parental mental health problems, 72 of them had two parents with mental health problems (8.9% of the total study population). The number and percentage of complete data are specified in S2 File.

**Table 1:** Participant characteristics of @ease visitors – 2018 to mid-2024.

Participant characteristics	*n* known*	*n* (%); mean (SD)
Age, years	840	20.3 (3.5)
Gender	888	
Female		547 (61.6%)
Male		317 (35.7%)
Non-binary		24 (2.7%)
Country of birth	874	
the Netherlands		523 (59.8%)
Other		351 (40.2%)
Country of birth	849	
the Netherlands		523 (61.6%)
Germany		63 (7.4%)
Other countries Europe		150 (17.7%)
Countries in Asia		65 (7.7%)
Countries in North America		21 (2.5%)
Countries in Africa		14 (1.7%)
Countries in South America		12 (1.4%)
Countries in Oceania		1 (0.1%)
Living situation	903	
Parent(s)		334 (37.0%)
Peers		235 (26.0%)
Alone		233 (25.8%)
Partner		40 (4.4%)
Assisted living		20 (2.2%)
Staying over		10 (1.1%)
Caregiver(s)		6 (0.7%)
Homeless		3 (0.3%)
Other		22 (2.4%)
Parental history of mental illness	802	
Mother		138 (17.2%)
Father		79 (9.9%)
Both parents		72 (8.9%)
None		513 (64.0%)
Daily occupation	881	
Education		609 (69.1%)
Job		94 (10.7%)
Education and job		72 (8.2%)
None		106 (12.0%)
Current education	756	
Primary school		15 (2.0%)
Secondary school		186 (24.6%)
Secondary vocational education		79 (10.5%)
Higher professional education		124 (16.4%)
University		352 (46.6%)
SOFAS score, mean (SD)	632	65.6 (14.6)

SD = standard deviation; * Categories do not have the same total scores because young people were allowed to skip questions.

### Health-related quality of life

@ease visitors presented with a mean utility of 0.64 (*SD* = 0.24). The EQ-5D-5L was complete in 758 questionnaires (80.6%) and ranged from 0.58 to 0.69 on average over the measured years (see Table 2). In 2020 and 2021, when the COVID-19 pandemic primarily took place, days of school absenteeism were lowest, and utilities were highest in 2020 (0.69).

**Table 2:** HRQoL, recent school absenteeism and recent mental healthcare throughout years, all within individual first visits to @ease.

	2018	2019	2020	2021	2022	2023	2024 – Jun
HRQoL (N) utilities, M (SD)	0.58 (0.23) (*n* = 28)	0.61 (0.24) (*n* = 102)	0.69 (0.22) (*n* = 95)	0.64 (0.25) (*n* = 146)	0.66 (0.24) (*n* = 145)	0.62 (0.22) (*n* = 150)	0.61 (0.29) (*n* = 92)
Mental healthcare use in past 3 months							
Yes	26 (47.3%) (*n* = 55)	34 (31.2%) (*n* = 109)	27 (26.2%) (*n* = 103)	38 (23.5%) (*n* = 162)	53 (32.5%) (*n* = 163)	68 (41.2%) (*n* = 165)	38 (40.4%) (*n* = 94)
Number of times, M (SD)	0.55 (1.27) (*n* = 38)	1.71 (5.07) (*n* = 108)	1.70 (4.73) (*n* = 101)	1.52 (7.58) (*n* = 159)	2.01 (5.57) (*n* = 160)	2.43 (5.10) (*n* = 153)	3.85 (10.87) (*n* = 91)
School absenteeism in past 3 months							
Yes	13 (54.2%) (*n* = 24)	53 (57.6%) (*n* = 92)	25 (30.9%) (*n* = 81)	35 (27.8%) (*n* = 126)	41 (26.3%) (*n* = 156)	57 (36.1%) (*n* = 158)	30 (35.3%) (*n* = 85)
Number of days, M (SD)	3.21 (5.08) (*n* = 24)	4.35 (8.54) (*n* = 91)	1.95 (5.24) (*n* = 81)	2.51 (6.58) (*n* = 126)	3.49 (11.19) (*n* = 154)	3.45 (9.78) (*n* = 158)	4.22 (10.75) (*n* = 85)

Note. *n* = number of times the question was answered that year; EQ-5D-5L was added to the questionnaire in August 2018.

The dimensions with the highest frequencies were usual activities (65.2% had any problems; 40.0% moderate or worse) and anxiety/depression (91.4% had any problems; 68.4% moderate or worse). Pain/discomfort caused problems for 48.6% of the young people, mostly at a minor to moderate level. Mobility and self-care were considered least problematic. Table 3 details the frequencies and proportions of the EQ-5D-5L dimensions, as recommended in the User Guide [23].

**Table 3:** Frequencies of responded levels on the five dimensions of the EuroQol (EQ-5D-5L) (n = 758).

	No problems N (%)	Minor problems N (%)	Moderate problems N (%)	Severe problems N (%)	Extreme problems N (%)
Mobility	726 (91.8)	46 (5.8)	16 (2.0)	2 (0.3)	1 (0.1)
Self-care	627 (79.4)	123 (15.6)	30 (3.8)	7 (0.9)	3 (0.4)
Usual activities	276 (34.8)	200 (25.2)	228 (28.8)	76 (9.6)	13 (1.6)
Pain/discomfort	405 (51.4)	200 (25.4)	135 (17.1)	36 (4.6)	12 (1.5)
Anxiety/depression	69 (8.7)	182 (23.0)	271 (34.3)	218 (27.6)	51 (6.5)

### Cost-of-illness

#### Mental healthcare costs.

On average, two mental healthcare consultations had taken place with a GP or psychologist in the three months prior to visiting @ease (see Table 4). This amounted to €234 on average per person in those three months among the *n* = 810 young people who answered frequency of care. Of the *n* = 851 persons who had answered *yes or no* to ‘having received care’, 284 (33.4%) indicated *yes*: they had minimally had one consultation in the last three months. Among the *n* = 243 consulters who reported their number of consultations, the average was 6.84 times (*SD* = 10.39), costing €781 (*SD* = €1,185). Table 2 shows the reported mean consultations in the past three months for each year, which was under one day in 2018, around two days in the years thereafter, and four days in the first half year of 2024.

**Table 4:** Resource use and costs in the 3 months prior to visiting @ease.

	*n* yes (%)	Unit	Units per person per 3 months, mean (SD)	Cost per person per 3 months, mean (SD)
Mental healthcare use (*n* = 851)	284 (33.4)	Contact	2.05 (6.49)	€234.18 (€740.57)
School absenteeism (*n* = 722)	254 (35.2)	Day	3.32 (9.06)	€358.03 (€824.90)
Both (*n* = 703)				€579.38 (€1083.91)

#### School absenteeism costs.

The average number of days of school absenteeism was 3.32 (*SD* = 9.06) in the last three months, amounting to a cost of €358 on average per student. Moreover, 247 visitors reported being absent from school at least one day. Among them, the average number of days was 9.60 (*SD* = 13.33) costing €983 (*SD* = €1,121). As shown in Table 2, the average number of days that young people were absent from school varied at around two to four days per three months over calendar years, with a peak of 4.35 days (*SD* = 8.54) on average in 2019. When looking into *academic* years, the average days of school absenteeism were 4.69 (*SD* = 9.32) in 2018–2019, followed by 2 to 2.5 days (*SDs* = 4.69–7.65) in the three years thereafter (2019–2022), and higher again in 2022–2023 at 4.08 days (*SD* = 12.49) and 3.83 (*SD* = 9.34) in 2023–2024 up to June.

#### Total costs.

The COI-questions were answered by *n* = 703 young people for *yes or no* and by *n* = 648 for the number of times. The summed costs for mental healthcare use and school absenteeism amounted to €375,436, which was €579 (*SD* = €1,084) per individual in the past three months. Per year, the sum of both COI-items amounted to €1,501,743, which was €2,318 per individual per year. Among those who answered both COI-questions, 312 (44.4%) young people had both not been absent from school and had zero mental health consultations in the three months prior to visiting @ease, i.e., made no such costs.

### Correlations prior to regressions

As specified in S3 File, number of mental healthcare visits and school absenteeism days correlated weakly and total financial burden and HRQoL utilities had a significant, inverted significant correlation with a weak strength. Independent and dependent variables correlated weakly with HRQoL utilities and negatively with total costs. Occupation correlated weakly with mental healthcare costs. Out of the five HRQoL-dimensions, problems with *usual activities* and *anxiety/depression* correlated negatively with the SOFAS, and problems with *usual activities* correlated positively with school absenteeism costs.

Correlations among the independent variables were negligible. Only country of birth correlated weakly with living situation and negatively with educational level. Among those born outside of the Netherlands, 37.6% were living alone and 85.2% were following a theoretical (not vocational) education. Among those born in the Netherlands, this was 18.2% and 55.8%, respectively.

### Predictors of HRQoL and cost-of-illness

HRQoL was positively predicted by social and occupational functioning (*β* = 0.01, *p* < .001). Conversely, living situation and parental mental health problems negatively predicted HRQoL, indicating a lower HRQoL for young people who were living alone (*β* = –0.06, *p* < .001) and who had parents with mental health problems (*β* = –0.05, *p* = .004), see Table 5.

**Table 5:** Multiple regression on predictors of utilities (EQ-5D-5L), school absenteeism costs, mental healthcare costs, and total costs (all post-imputation).

	Utilities (EQ-5D-5L)			
	*B*	S.E.	*t*	p *
(Constant)	0.28	0.05	5.96	**<.001**
Gender (Female)	−0.04	0.02	−2.29	.022
Gender (Non-binary)	−0.13	0.05	−2.37	.018
Living situation (Alone)	−0.06	0.02	−3.42	**<.001**
Social and occupational functioning	0.01	0.00	9.42	**<.001**
Parental mental health problems	−0.05	0.02	−2.90	**.004**
COVID-19 pandemic	0.03	0.02	1.82	.069
Occupation (None)	0.02	0.03	0.73	.463
	**School absenteeism costs**			
	***B***	**S.E.**	***t***	p *
(Constant)	342.23	65.18	5.25	**<.001**
Gender (Male)	8.46	67.27	0.13	.900
Living situation (Alone)	106.74	78.21	1.37	.172
Parental mental health problems	68.35	67.47	1.01	.311
Country of birth (Not the Netherlands)	223.39	69.61	3.21	**.001**
Education (Vocational)	33.15	84.87	0.39	.696
COVID-19 pandemic	−188.05	69.94	−2.69	**.007**
	**Mental healthcare costs**			
	***B***	**S.E.**	***t***	p *
(Constant)	317.64	59.79	5.31	**<.001**
Gender (Female)	−31.78	50.78	−0.63	.532
Living situation (Alone)	37.89	58.22	0.65	.515
School absenteeism	54.95	52.53	1.05	.296
Education (Vocational)	130.31	71.44	1.82	.068
Country of birth (Not the Netherlands)	−137.00	54.30	−2.52	**.012**
Parental mental health problems	28.20	52.14	0.54	.589
COVID-19 pandemic	−136.75	53.85	−2.54	**.011**
Occupation (None)	470.44	99.19	4.74	**<.001**
	**Total costs**			
	***B***	**S.E.**	***t***	p *
(Constant)	877.16	112.21	7.82	**<.001**
Gender (Female)	−23.26	87.11	−0.27	.789
Gender (Non-binary)	136.96	250.78	0.55	.585
Living situation (Alone)	212.04	99.43	2.13	.033
Country of birth (Not the Netherlands)	129.12	89.13	1.45	.148
Education (Theoretical)	−292.69	106.19	−2.76	**.006**
Parental mental health problems	109.37	87.51	1.25	.211
COVID-19 pandemic	−238.04	89.70	−2.65	**.008**

Note. All costs in Euros (€); * With Bonferroni correction, the threshold for statistical significance was a = .013.

School absenteeism costs were positively predicted by country of birth and negatively by the COVID-19 pandemic, indicating that school absenteeism costs were higher for those born outside of the Netherlands (*β =* 223.39, *p* = .001) and lower for those who visited during the COVID-19 pandemic (*β =* –188.05, *p* = .007). Mental healthcare costs were positively predicted by occupation, indicating higher care costs for young people who had no occupation (i.e., who were not following an education or working) (*β =* 470.44, *p* < .001), and negatively by country of birth and the COVID-19 pandemic, indicating lower mental healthcare care costs for those born outside of the Netherlands (*β =* –137.00, *p* = .012) and those visiting during the COVID-19 pandemic (*β =* –136.75, *p* = .011). Total COI was negatively predicted by education and visiting during the COVID-19 pandemic, suggesting lower total financial burden for those studying at a theoretical education (*β =* –292.69, *p* = .006) and visiting during the COVID-19 pandemic (*β =* –238.04, *p* = .008). All regression results are specified in Table 5, all at *a* = .013.

The complete cases analysis (See S4 File) also resulted in social and occupational functioning being a significant positive predictor of HRQoL. Country of birth was again a positive predictor of school absenteeism costs and occupation was again a positive predictor of mental healthcare costs, while total costs were positively predicted by living situation and negatively by education.

### Bootstrapped subgroup analyses of cost-of-illness

The bootstrapped costs and differences between costs within subgroups are shown in Table 6. Young people born outside of the Netherlands or visiting outside of the COVID-19 pandemic had significantly higher school absenteeism costs, and young people without an occupation or born in the Netherlands had significantly higher mental healthcare costs than their peers. No subgroups statistically significantly differed in total COI.

**Table 6:** Sub-group analyses of cost-of-illness in the past three months (post-imputation).

		Costs per person	Bootstrapped costs per person	Bootstrapped difference	
	N	Mean (SD)	Mean (SD)	Mean	95% CI*
**(a) Sub-group analyses of school absenteeism costs**					
Male and female				51.99	−143.57 to 288.09
Male	257	460.25 (823.56)	319.61 (43.17)		
Female	532	433.83 (752.23)	371.60 (41.90)		
Non-binary and male				−408.35	−1.296.00 to 203.69
Non-binary	22	722.69 (1244.52)	726.64 (262.31)		
Male	257	460.25 (823.56)	318.29 (42.09)		
Non-binary and female				−354.39	−1.353.23 to 287.67
Non-binary	22	722.69 (1244.52)	723.51 (256.39)		
Female	532	433.83 (752.23)	369.12 (40.90)		
Living situation				−177.73	−429.33 to 66.75
Alone	193	585.27 (948.34)	585.15 (65.89)		
Other	618	407.81 (732.57)	407.42 (41.26)		
Parental history of mental illness				−9.00	−254.02 to 203.74
Yes	255	453.29 (841.82)	456.78 (52.62)		
No	556	448.55 (769.26)	447.78 (44.62)		
Born in Netherlands				276.04**	101.72 to 469.20**
Yes	468	348.64 (689.37)	289.99 (37.65)		
No	343	588.40 (896.90)	566.03 (52.26)		
Education				−78.57	−339.08 to 298.86
Theoretical	666	464.12 (777.42)	466.91 (46.90)		
Vocational (VMBO/MBO)	130	387.63 (853.58)	388.34 (74.64)		
During pandemic				235.80**	49.49 to 432.70**
Yes	226	281.96 (552.44)	280.24 (37.01)		
No	585	514.97 (858.99)	516.04 (48.66)		
**(b) Sub-group analyses of mental healthcare costs**					
Male or female				−78.00	−316.87 to 103.10
Male	323	353.26 (967.01)	352.90 (56.79)		
Female	592	274.98 (531.33)	274.91 (29.92)		
Non-binary and male				141.42	−116.96 to 484.27
Non-binary	25	213.85 (304.93)	214.47 (59.76)		
Male	323	353.26 (967.01)	355.89 (56.09)		
Non-binary and female				61.29	−144.90 to 233.14
Non-binary	25	213.85 (304.93)	214.57 (59.39)		
Female	592	274.98 (531.33)	275.86 (31.14)		
Living situation				−5.64	−189.62 to 199.62
Alone	237	307.37 (534.89)	306.51 (34.68)		
Other	703	297.86 (758.72)	300.87 (44.14)		
Occupation ^a^				460.48**	39.26 to 1,041.30**
Yes	873	267.69 (614.21)	266.61 (34.95)		
No	67	724.57 (1401.85)	727.08 (179.92)		
Parental history of mental illness				−26.39	−199.47 to 164.54
Yes	302	318.52 (620.33)	317.67 (35.61)		
No	638	291.61 (747.23)	291.27 (44.44)		
Born in Netherlands				−183.77**	−369.44 to −25.38**
Yes	576	371.30 (844.69)	370.73 (50.43)		
No	364	187.83 (385.26)	186.96 (22.84)		
Education				180.10	−5.95 to 460.06
Theoretical	666	220.92 (398.54)	222.28 (23.38)		
Vocational (VMBO/MBO)	130	404.14 (789.42)	402.38 (69.93)		
School absenteeism in past 3 months				−68.14	−212.71 to 119.34
Yes	374	304.74 (490.39)	304.33 (28.22)		
No	437	238.67 (707.15)	236.18 (41.00)		
During pandemic				118.47	−91.15 to 347.35
Yes	273	214.84 (740.61)	215.62 (45.58)		
No	667	335.21 (692.79)	334.09 (39.71)		
**(c) Sub-group analyses of total costs if both items were filled out**					
Male or female				−81.89	−416.90 to 286.53
Male	257	769.69 (1,166.86)	769.78 (73.71)		
Female	532	686.33 (961.33)	687.89 (53.72)		
Non-binary and male				−136.97	−1,312.71 to 560.42
Non-binary	22	904.11 (1216.40)	908.34 (250.32)		
Male	257	769.69 (1166.86)	771.36 (72.35)		
Non-binary and female				−201.76	−1,066.18 to 398.24
Non-binary	22	904.11 (1216.40)	887.48 (249.49)		
Female	532	686.33 (961.33)	685.72 (54.32)		
Living situation				−228.25	−572.62 to 103.26
Alone	193	889.76 (1135.24)	891.32 (81.14)		
Other	618	665.22 (1000.39)	663.07 (57.82)		
During pandemic				283.07	−44.90 to 576.75
Yes	226	512.61 (1020.21)	514.75 (66.59)		
No	585	798.25 (1034.38)	797.82 (58.89)		
Parental history of mental illness				−32.18	−279.85 to 207.57
Yes	255	741.20 (1036.02)	743.95 (63.51)		
No	556	708.31 (1039.34)	711.78 (60.31)		
Born in Netherlands				97.00	−159.14 to 453.46
Yes	468	676.59 (1032.06)	678.02 (61.69)		
No	343	776.05 (1044.27)	775.03 (59.58)		
Education				105.78	−283.25 to 523.16
Theoretical	666	684.39 (905.30)	682.54 (51.64)		
Vocational (VMBO/MBO)	130	791.76 (1197.81)	788.32 (101.14)		

Note. All costs in Euros (€); SD = standard deviation; CI = confidence interval; If CI includes 0, no significant difference is found; ** Significant difference.

## Discussion

This study provided insight into the burden of mental health problems among 940 young people by presenting their HRQoL and COI when first accessing walk-in peer counselling at @ease in the Netherlands, supplementing an earlier study [17] that this work followed up on.

The previous [17] and present study showed that young people who sought peer support at @ease reported a lower HRQoL (0.62 then, 0.64 now) compared with the HRQoL of youth in the general Dutch population (0.91–0.96) [24], experiencing anxiety and depressive feelings to a greater extent than their peers. Over one-third of @ease visitors had been absent from school in the past three weeks, compared to national findings among youth aged 16–25 years of 5.2% having been truant (3 + hours) and 18.8% having had sick days (3 + days), as measured over the past month [35]. Furthermore, in the present study, one-third had received mental healthcare in the past three months. Combined, all costs amounted to an annual COI of over 1.5 million euros. To prevent (further) impairment and higher societal costs, it is crucial to improve early detection and invest in interventions for youth mental health. Present findings show links between mental health factors and indicate risk groups for a higher burden of disease.

The found connection between quality of life and social and occupational functioning is consistent with the first study [17] which this study is a continuation of, and with other contexts of, e.g., remitted psychosis [36], risk of psychosis [37], and depressive disorders [38]. These constructs may form a downward spiral; first, maladaptive emotional and social awareness and regulation tend to lead to isolation and social problems, in turn lowering mood through processes such as rumination about rejection, becoming a vicious cycle [39,40]. Likewise, for occupational functioning, reduced pleasure may bring about lower performance, again inducing less satisfaction [41]. In the opposite direction, this offers opportunities in practice to strengthen meaningful relationships and/or professional effectivity at work or school to lower anxiety and sadness, in turn increasing engagement in usual activities.

Similarly consistent with literature were our findings that a lower quality of life was observed when living alone [17,42] or having parents with mental health problems [43,44]. However, living alone might not relate directly to poor mental health, as loneliness may be the underlying factor [45,46]. Furthermore, while parental mental health problems have been associated with lower HRQoL, social resources outside of the family were earlier identified as significant resources [44,47]. As such, visiting @ease for peer support may aid in improving the HRQoL of these young people.

Present findings also indicate that young people who had not been working or studying had significantly more care costs for mental health or addiction-related problems, but not a significantly lower HRQoL. Young people without an occupation might have accessed care for reasons unrelated to the mood and anxiety enquired within HRQoL, such as attention deficits, or addiction without comorbidity with mood or anxiety. Additionally, their number of consultations might have been higher due to less time constraints when being at home, or higher pressure to return to work or education. Finding no significant link with HRQoL is still surprising, as the ability to do usual activities may be lower, and as unemployed individuals in the Netherlands were found to have any mental disorder more often than those employed or studying [15]. Among young people, reasons for not having an occupation may vary too much to consistently predict low HRQoL, from consciously choosing a gap year to travel, to being at home unwillingly with a condition that can influence HRQoL. Having an occupation may on the other hand introduce a burden of responsibilities and expectations that influences HRQoL, as @ease visitors have indicated in practice, but not per se lead to seeking specialized help.

Unexpectedly, the COVID-19 pandemic did not predict HRQoL while seeking peer support. Most earlier studies have shown higher symptoms of anxiety and depression among youth during the COVID-19 pandemic, as concluded in a large systematic review [48]. However, this study applied the full timeframe from the official declaration of a pandemic on 11^th^ of March 2020 up to the lifting of all measurements starting March 2022 [49], not inspecting specific spikes in COVID-19 cases or governmental measures. Moreover, the HRQoL domains *Usual activities* and *Self-care* might have been rated higher during the COVID-19 pandemic due to fewer activities and a different definition of what was normal at the time.

The lower school absenteeism costs found during the COVID-19 pandemic may explain why the average number of days of school absenteeism in the original pre-pandemic study [17] was one day higher compared to the presently fuller timespan. Online and hybrid education during the pandemic may have made it easier to attend classes. However, being present online does not inform us about the level of productivity, i.e., less absenteeism might originate from a lower threshold to attend digitally. Furthermore, mental healthcare costs may have been lower during the COVID-19 pandemic if more young people preferred not to receive digital treatment when face-to-face therapy was not optional or posed a health risk.

The estimated costs were higher depending on country of birth, but in opposite directions; school absenteeism costs were higher and mental healthcare costs were lower for young people born outside of the Netherlands. As reasoned in the original study by Leijdesdorff et al. [17], not speaking the Dutch language and being unfamiliar with the route to care can hinder seeking and receiving mental healthcare. Furthermore, overrepresentation of university students that exists due to @ease having started up in larger cities, might be reflected in the school absenteeism figures, as attendance is often not obligatory for all classes in tertiary education. More indicated missed classes might then not have as large of an impact as for primary and secondary education.

Various other predictors or subgroups were expected from literature but were not found to be significant in this population, which might stem from differences in definitions and measurements. For example, earlier studies described in Boonstra et al. [20] had tested, e.g., lower academic achievement and lower IQ as predictors of school absenteeism, which could deviate too much from the educational level which was tested as a predictor in this study.

Total COI was €2,318 per individual per year, whereas this was €2,051 in the previous study [17]. This difference mostly exists due to the number of days that mental healthcare was accessed on average in the past three months, which was one day then and two days now, doubling these costs. To reverse an increasing need for mental healthcare use, and with that the high pressures on and shortages among GPs and mental health professionals [50,51], prevention is of high importance.

No significant predictors were found for total COI. Yet, Leijdesdorff et al. [17] had found earlier that living alone was a significant predictor of COI among @ease visitors. In both studies, the directionality of the findings was the same and originated from school absenteeism costs. The finding that the difference between those living alone or not was much larger in the previous study might be explained by the fact that the number of participants was relatively low then (*n* = 21 living alone), and not all were necessarily studying. Furthermore, the average days of school absenteeism were higher in the previous study, at four days instead of three. As mentioned, this seemingly improved attendance appears to be influenced by the time of digital and hybrid education during the COVID-19 pandemic, which had not yet occurred in the previous study.

### Implications and further research

Young people who sought peer support at @ease indicated that they did so to address personal feelings, social relationships, and education/work. These needs are reflected in the relatively low HRQoL and social and occupational functioning. To empower young people in their functioning and to validate feelings, peer counsellors actively listen and apply trained motivational interviewing and solution-focused strategies [19]. Social support is an important predictor of quality of life among young people [52], which is provided by the peer counsellors but also stimulated by working on improving social contacts and support in daily life. Peer support offers a unique opportunity for self-disclosure about coping strategies and meaningful activities from a counsellor in a similar age group. While HRQoL has yet to be analysed over time in @ease visitors, social and occupational functioning improved significantly over @ease visits [18].

It is unknown whether school absenteeism is often addressed in @ease peer counselling. It was found that around one-third had been absent from school in the past three months, hence, the peer counsellors can be motivated in the trainings and by the on-site professional to address this topic in peer counselling with the aim to help prevent both educational disadvantages such as learning delays, as well as costs. However, working on areas brought up by the young person might indirectly help solve reasons underlying missed attendance.

For most visitors, @ease had been the first form of mental health support in the past three months; 31% of all visitors had recently visited a psychologist or GP for mental health or addiction-related problems, while 69% scored moderately to severely on the domain of anxiety/depression. It is yet unknown whether @ease counselling prevents young people from requiring further care, thereby preventing costs. However, 72% of one-time visitors and 81% of returnees indicated that they did not plan to go to the GP or a psychologist after @ease counselling [18]. In addition, satisfaction with @ease was found to be high [18,19]. Hence, for some young people, consulting @ease might be sufficient at that particular timepoint to prevent worsening, whilst others are offered support in finding fitting care, also to avoid problems in accessing (the wrong) services and to prevent unneeded costs. New research is devoted to the evaluation of the cost-effectiveness of @ease, through the Europe-wide research project called YOUTHreach (https://youth-reach.eu/) [53].

### Strengths and limitations

This study uniquely showed the burden of mental health problems among 940 young people in the Netherlands, as well as potential risk groups. Strengths in the design of this study include the bottom-up process, which provides less biased estimates than a top-down approach [54], and the societal perspective, optimal for measuring impact and making decisions [55]. However, it must be emphasized that societal costs are likely higher, as school absenteeism can also result in, e.g., police costs and costs of social workers or costs for family members, and care use costs are likely higher in general due to, e.g., comorbidity and somatisation. @ease is dedicated to providing accessible support and, as a part of that, questionnaires are kept short and anonymous. As a by-product, these questionnaires might miss details such as reasons for school absenteeism or having no occupation, or the form of received care. If we could distinguish between those happily (e.g., due to a gap year) and unwillingly having no occupation (e.g., due to mental or physical illness), perhaps differences in HRQoL would be found and could be explained further. This would align with the lower HRQoL found among adults without an occupation, where unemployment might more-often be unwillingly. Context is also missing regarding parental mental health problems. Beside the yes/no answer, young people wrote in the open-text field, e.g.,: *“I think mom had a depression once”* or *“dad drinks a lot”*, but it is unknown whether young people experienced their family situation as influential to their wellbeing. Besides detailed predictors, we might miss costs of, e.g., job absenteeism and other forms of care. As such, the questionnaire is being updated to more fully measure cost-effectiveness, while keeping it as short as possible.

A longer questionnaire might be less biased due to providing more detailed information, but its length must remain feasible to avoid biases through, e.g., response fatigue or disinclination to complete questionnaires. Presently, 19% of EQ-5D-5L data was missing already and it is unknown whether this stemmed from, e.g., fatigue as this survey was also at the end of the questionnaire, disinterest, or eagerness to leave, or reasons that could contribute to underestimation of results, such as symptom severity. While multiple imputation reduces bias due to item‑level missingness among respondents, it cannot correct for potential systematic differences between respondents and non‑respondents.

Results may not be generalizable across all young people, as almost half of the participants were studying at a university, many were not born in the Netherlands, and most were living alone or with peers. @ease started up in cities with large universities, however, this is expected to become more balanced in future studies with newer walk-in centres expanding. In addition, results should be read in the context of young people at-risk of, or experiencing, mental health problems, as all participants were help-seeking in the sense that they visited @ease for peer support, and their HRQoL was worse than in the general population.

While @ease is centred on high accessibility for help-seeking, both practically and in terms of destigmatizing and youth-orientation, not all who need support may access it. Hence, young people could be missing from this data. For example, not all young people may be able to travel to a walk-in centre. To reach young people at a distance, @ease also travelled into neighbourhoods [56], offers an online chat service [57], and is spreading to more locations. However, if problems are so severe that the step to seek help is too large, or if one is overburdened and makes no time for visiting, we might miss data on young people for whom the burden is on the higher end. Conversely, young people who visited @ease sometimes indicated that they had not visited earlier due to fears that problems may not be large or important enough, or that others may judge if they find out about their help-seeking, which may thereby also be underrepresented groups in the data or groups that find their way to peer support later.

## Conclusion

Present findings signalled risk groups with a higher burden of disease among young people accessing peer support for mental health (and related) problems. Early detection of such risk groups and investment in early intervention are of great importance to prevent (further) impairment and higher societal costs. Additionally, to lower the burden, interventions may target social and occupational functioning and provide support regarding contextual risk factors. More prospective effectiveness and health-economic evaluations are needed.

## Supporting information

S1 File Calculation of school absenteeism costs and mental health care use [27,58,59]. (DOCX)

S2 File Number and percentage of complete data before multiple imputation. (DOCX)

S3 File Correlation matrix. (DOCX)

S4 File Complete case analysis. (DOCX)

S5 File Consensus-based checklist for COI studies. (DOCX)
