# Supplementary material for: Health-related quality of life and cost-of-illness in young people seeking peer support at @ease: A Dutch burden of disease study
Source: PLoS One. 2026 Jul 6;21(7):e0352652. doi: 10.1371/journal.pone.0352652 (PMC13336155; doi:10.1371/journal.pone.0352652)
Supplement: S1 File — (DOCX) [file pone.0352652.s001.docx]

**S1 File. Calculation of school absenteeism costs and mental health care use.**

| **Supplementary Table 1. Educational expenses of 2020 with inflation correction to 2024, in euros (€).** | | | | | |
| --- | --- | --- | --- | --- | --- |
|  | **Number of students** | **Total expenses ^a^** | **Per person** | **Per person per day ^d^** | **Inflation correction ^e^** |
| Education level |  |  |  |  |  |
| (Pre)primary education ^b^ | 1421800 | € 13,173,000,000 | € 9,265 | **€ 46** | **€ 55** |
| Secondary general education | 934200 | € 11,897,000,000 | € 12,735 | **€ 64** | **€ 76** |
| Senior voc. and gen. adult sec. education ^c^ | 508800 | € 7,765,000,000 | € 15,261 | **€ 76** | **€ 91** |
| Higher professional education | 489300 | € 6,264,000,000 | € 12,802 | **€ 64** | **€ 77** |
| University | 331400 | € 8,707,000,000 | € 26,273 | **€ 131** | **€ 157** |
| Sources: Statistics Netherlands (2023a; 2023b).  **^a^** For every category of expenses *(government, households, companies, non-profit organizations and foreign countries)*, the receipts were subtracted from the expenses to obtain expenses only.  Whilst subtracting ‘*government: receipts’* from the total, the ‘repayments on student loans’ were not subtracted as these cancel out due to payment and repayment. Whilst subtracting ‘*households: allowances received’* from the total, the ‘Student grants for the cost of living’ and ‘Student loans’ were not subtracted for the same reason.  Lastly, whilst subtracting ‘*companies: subsidies received’* from the total, ‘transport of pupils’ was not subtracted.  ^b^ Basisonderwijs en speciaal basisonderwijs opgeteld  ^c^ Senior vocational and general adult secondary education  ^d^ Cost per day was calculated by dividing the cost per person per year by 200 school days, based on 40 weeks and 5 days per week, consistent with the previous paper (Leijdesdorff et al., 2020) and based on the method used by Drost et al. [58].  ^e^ Inflation correction with a factor of *1.29 (CPI 107.51% by 2020 to CPI 128.77% by June 2024) | | | | | |

| **Supplementary Table 2. Percentage of care use (GP and GGZ) and prices per contact, in euros (€).** | | |
| --- | --- | --- |
|  | **General practitioner (GP)** | **GGZ** |
| % care use among people with mental disorder(s) ^a^ | 28.3% | 23.0% |
| % care use in general population | 10.9% | 8.8% |
| Price in euros (€) of one contact ^b^ | €62 | €164 ^c^ |
| Source: NEMESIS-3 (2019-2022) and Hakkaart-van Roijen et al. [27]  ^a^ derived from “Enige psychische stoornis stoornis”  ^b^ At the GP, Hakkaart-van Roijen et al. [59] stated that typically a ‘double consult’ is booked for patients with mental problems. Hence, we doubled €30,87 (GP average consult price) from Hakkaart-van Roijen et al. [27] as in the previous study.  ^c^ Average of a consult with: “*Vrijgevestigd zorgverlener in de basis GGZ*”, “*Generalistische basis GGZ-instellingen, contact zorgverlener*”, “*Contact vrijgevestigd zorgverlener in de specialistische GGZ*” and “*contact zorgverlener in de specialistische GGZ*”, also including “*verpleegdag psychiatrische instelling*” | | |

| **Supplementary Table 3. Weighted average contact cost using percentage of care use and prices in euros (€).** | | | | |
| --- | --- | --- | --- | --- |
|  | **With mental disorder(s) ^a^** | **In general population ^a^** | **Average** | **Average with inflation correction ^b^** |
| Weighted average of GP and GGZ price (€) and % of use | €107.64 | €107.47 | €107.56 | €114 |
| ^a^ Calculated by: ((GP % * GP price) + (GGZ % * GGZ price)) / (GP % + GGZ %)  ^b^ Inflation correction with a factor of *1.06 (CPI 121.43% by 2022 to CPI 128.77% by June 2024) | | | | |
