# Supplementary material for: Health-related quality of life and cost-of-illness in young people seeking peer support at @ease: A Dutch burden of disease study
Source: PLoS One. 2026 Jul 6;21(7):e0352652. doi: 10.1371/journal.pone.0352652 (PMC13336155; doi:10.1371/journal.pone.0352652)
Supplement: S2 File — (DOCX) [file pone.0352652.s002.docx]

**S2 File. Number and percentage of complete data before multiple imputation.**

| **Supplementary Table 4. Number and percentage of participants with complete data (n=940).** | |
| --- | --- |
|  | Complete data *n* (%) |
| Outcome measures |  |
| HRQoL (EQ-5D-5L) | 758 (81%) |
| School absenteeism costs | 673 (72%) |
| Mental healthcare costs | 810 (86%) |
| Total costs (both categories known) | 648 (69%) |
| Tested predictors |  |
| Gender | 888 (94%) |
| Occupation and/or education* | 885 (94%) |
| Living situation | 903 (96%) |
| Parental mental health problems | 802 (85%) |
| Country of birth** | 874 (93%) |
| COVID-19 pandemic | 940 (100%) |
| School absenteeism | 722 (77%) |
| Social and occupational functioning (SOFAS) | 632 (67%) |
| * “Occupation and/or education” was later dummy coded into Occupation (1=None) and Education (1=vocational or theoretical depending on the test), ** The n of “Country of birth” differs with its n in the characteristics table because there the open text-field was used that specifies the country, whereas the binary question (The Netherlands or Other) noted here was used for the analyses. | |
