# Supplementary material for: Health-related quality of life and cost-of-illness in young people seeking peer support at @ease: A Dutch burden of disease study
Source: PLoS One. 2026 Jul 6;21(7):e0352652. doi: 10.1371/journal.pone.0352652 (PMC13336155; doi:10.1371/journal.pone.0352652)
Supplement: S3 File — (DOCX) [file pone.0352652.s003.docx]

**S3 File. Correlation matrix.**

| **Supplementary Table 5. Correlation matrix (post-imputation).** | | | | | | | | | | | | | | | | | |
| --- | --- | --- | --- | --- | --- | --- | --- | --- | --- | --- | --- | --- | --- | --- | --- | --- | --- |
|  | **Variable** | **1** | **2** | **3** | **4** | **5** | **6** | **7** | **8** | **9** | **10** | **11** | **12** | **13** | **14** | **15** | **16** |
| 1 | Utility (EQ-5D-5L) | . |  |  |  |  |  |  |  |  |  |  |  |  |  |  |  |
| 2 | Mental healthcare costs | -0.13*** | . |  |  |  |  |  |  |  |  |  |  |  |  |  |  |
| 3 | School absenteeism costs | -0.18*** | 0.06 | . |  |  |  |  |  |  |  |  |  |  |  |  |  |
| 4 | Total costs | -0.22*** | . | 0.81*** | . |  |  |  |  |  |  |  |  |  |  |  |  |
| 5 | Gender (male) | 0.06 | 0.05 | 0.00 | 0.02 | . |  |  |  |  |  |  |  |  |  |  |  |
| 6 | Gender (female) | -0.03 | -0.05 | -0.02 | -0.03 | . | . |  |  |  |  |  |  |  |  |  |  |
| 7 | Gender (non-binary) | -0.10* | -0.02 | 0.05 | 0.03 | . | . | . |  |  |  |  |  |  |  |  |  |
| 8 | Living situation | -0.12*** | 0.01 | 0.10* | 0.10** | 0.07* | -0.08* | 0.02 | . |  |  |  |  |  |  |  |  |
| 9 | Country of birth | -0.12*** | -0.11*** | 0.15*** | 0.06 | -0.07* | 0.06 | 0.04 | 0.22*** | . |  |  |  |  |  |  |  |
| 10 | COVID-19 Pandemic | 0.09** | -0.08* | -0.13*** | -0.12*** | -0.03 | 0.03 | -0.02 | -0.09** | -0.11*** | . |  |  |  |  |  |  |
| 11 | Parental mental health problems | -0.14*** | 0.04 | 0.03 | 0.05 | -0.03 | 0.02 | 0.05 | 0.01 | -0.11** | -0.06 | . |  |  |  |  |  |
| 12 | School absenteeism days | -0.20*** | 0.04 | . | . | -0.01 | 0.01 | 0.02 | 0.02 | 0.13*** | -0.14*** | 0.05 | . |  |  |  |  |
| 13 | Mental healthcare visits | -0.23*** | . | 0.08* | . | 0.02 | -0.02 | 0.01 | 0.05 | -0.05 | -0.13*** | 0.09** | 0.13*** | . |  |  |  |
| 14 | Education | 0.08* | 0.06 | -0.02 | 0.04 | 0.09** | -0.08* | -0.04 | -0.10** | -0.22*** | -0.00 | 0.06 | -0.03 | 0.04 | . |  |  |
| 15 | Occupation | -0.03 | 0.17*** | ^a^ | ^a^ | 0.06 | -0.07 | 0.01 | 0.04 | -0.15*** | 0.06 | 0.03 | ^a^ | 0.12*** | -0.12*** | . |  |
| 16 | Social and occupational functioning (SOFAS) | 0.38*** | -0.17*** | -0.17*** | -0.26*** | -0.07 | 0.08* | -0.05 | -0.01 | 0.06 | 0.04 | -0.09* | -0.14*** | -0.22*** | -0.05 | -0.14*** | . |
| Note. * *p* ≤ .05, ** *p* ≤ .01, *** *p* ≤.001. | | | | | | | | | | | | | | | | | |

|  |
| --- |
