# Supplementary material for: Health-related quality of life and cost-of-illness in young people seeking peer support at @ease: A Dutch burden of disease study
Source: PLoS One. 2026 Jul 6;21(7):e0352652. doi: 10.1371/journal.pone.0352652 (PMC13336155; doi:10.1371/journal.pone.0352652)
Supplement: S4 File — (DOCX) [file pone.0352652.s004.docx]

**S4 File. Complete case analysis.**

| **Supplementary Table 6. Multiple regression - Complete case analysis.** | | | | |
| --- | --- | --- | --- | --- |
|  | **Utilities (EQ-5D-5L)** | | | |
|  | ***B*** | ***S.E.*** | ***t*** | ***p **** |
| (Constant) | 0.26 | 0.05 | 5.02 | **<.001** |
| Gender (Female) | -0.04 | 0.02 | -1.72 | .086 |
| Gender (Non-binary) | -0.10 | 0.05 | -1.92 | .056 |
| Living situation (Alone) | -0.05 | 0.02 | -2.04 | **.**042 |
| Social and Occupational Functioning | 0.01 | 0.00 | 9.09 | **<.001** |
| Parental mental health problems | -0.05 | 0.02 | -2.47 | **.**014 |
| COVID-19 pandemic | 0.02 | 0.02 | 0.84 | .403 |
| Occupation (None) | 0.05 | 0.04 | 1.15 | .251 |

|  | **School absenteeism costs** | | | |
| --- | --- | --- | --- | --- |
|  | ***B*** | **S.E.** | ***t*** | ***p **** |
| (Constant) | 254.77 | 70.32 | 3.62 | **<.001** |
| Gender (Male) | 26.33 | 71.91 | 0.37 | .714 |
| Living situation (Alone) | 169.25 | 82.53 | 2.05 | .041 |
| Parental mental health problems | 59.88 | 70.44 | 0.85 | .396 |
| Country of birth (Not The Netherlands) | 185.16 | 73.27 | 2.53 | **.012** |
| Education (Vocational) | 29.78 | 91.92 | 0.32 | .746 |
| COVID-19 pandemic | -150.88 | 73.77 | -2.05 | .041 |

|  | **Mental healthcare costs** | | | |
| --- | --- | --- | --- | --- |
|  | ***B*** | **S.E.** | ***t*** | ***p **** |
| (Constant) | 279.89 | 68.72 | 4.07 | **<.001** |
| Gender (Female) | -17.95 | 59.27 | -0.30 | .762 |
| Living situation (Alone) | 29.14 | 68.60 | 0.42 | .671 |
| School absenteeism | 14.09 | 64.37 | 0.22 | .827 |
| Education (Vocational) | 141.82 | 83.70 | 1.69 | .091 |
| Country of birth (Not The Netherlands) | -141.59 | 63.46 | -2.23 | .026 |
| Parental mental health problems | 15.37 | 60.19 | 0.26 | .799 |
| COVID-19 pandemic | -119.88 | 62.07 | -1.93 | .054 |
| Occupation (None) | 473.67 | 123.35 | 3.84 | **<.001** |

|  | **Total costs** | | | |
| --- | --- | --- | --- | --- |
|  | ***B*** | **S.E.** | ***t*** | ***p **** |
| (Constant) | 773.66 | 125.46 | 6.17 | <.001 |
| Gender (Female) | -28.19 | 97.07 | -0.29 | .772 |
| Gender (Non-binary) | 287.54 | 277.80 | 1.04 | .301 |
| Living situation (Alone) | 310.35 | 110.63 | 2.81 | **.005** |
| Country of birth (Not The Netherlands) | 77.49 | 97.27 | 0.80 | .426 |
| Education (Theoretical) | -323.22 | 118.37 | -2.73 | .**007** |
| Parental mental health problems | 77.32 | 94.14 | 0.82 | .412 |
| COVID-19 pandemic | -160.69 | 98.31 | -1.63 | .103 |
| ** With Bonferroni correction, the threshold for statistical significance was a=.013.* | | | | |
