# Supplementary material for: Health-related quality of life and cost-of-illness in young people seeking peer support at @ease: A Dutch burden of disease study
Source: PLoS One. 2026 Jul 6;21(7):e0352652. doi: 10.1371/journal.pone.0352652 (PMC13336155; doi:10.1371/journal.pone.0352652)
Supplement: S5 File — (DOCX) [file pone.0352652.s005.docx]

**S5 File. Consensus-based checklist for COI studies.**

| **Supplementary Table 7. Consensus-based checklist for COI studies.** | | | |
| --- | --- | --- | --- |
| **Item** | **Question** | **Answer*** | **Supportive information** |
| Study characteristics |  |  |  |
| Question/objective | 1) Is a well-defined research question or objective stated? | Yes | See end of *Introduction, page 3-4.* |
| Population | 2) Is the study population described? | Yes | See *Results* and *Table 1, page 6-7.* Clinical characteristics are not applicable. |
| Perspective | 3) a) Is (are) the chosen study perspective(s) stated? | Yes | See end of *Introduction,* and *Study design and setting, page 4.* |
|  | b) If so, is (are) the chosen study perspective(s) justified? | Yes | See *Study design and setting, page 4.* |
| Methodology and cost analysis | | | |
| Epidemiological approach | 4) Is the epidemiological approach reported (e.g., prevalence, incidence)? | Yes | See *Study design and setting, page 4.* |
| Costing approach | 5) Is the costing approach reported (e.g., top-down, bottom-up)? | Yes | See *Study design and setting, page 4.* |
| Data collection approach | 6) Is the data collection process reported (e.g., prospective, retrospective)? | Yes | See *Study design and setting, page 4.* This study was cross-sectional, one measurement per participant, and costs were enquired retrospectively over the past three months. |
| Identification | 7) a) Are all components of resource use identified that are relevant to the condition/disease, population, intervention, study objectives, and study perspective? | Partially | See *Study design and setting, page 4,* and *Discussion: Strengths and limitations, page 18.* For optimal accessibility, a brief questionnaire was chosen that does not include every possible cost component that could be relevant in this population. |
|  | b) If not, is a justification provided for excluding relevant components of resource use? | Yes | See *Study design and setting, page 4,* and *Discussion: Strengths and limitations, page 18.* |
| Measurement | 8) a) Are all included components of resource use measured? | Yes | The questionnaire was co-developed with young people to be tailored to the population, and brevity was one priority expressed by young people; therefore, costs were single items at present. |
|  | b) If not, is a justification provided for not measuring certain components of resource use? | Yes | For optimal accessibility, a brief questionnaire was chosen that does not include every possible cost component that could be relevant in this population, see *Study design and setting, page 4,* and *Discussion: Strengths and limitations, page 18.* |
| Valuation | 9) a) Are all included components of resource use valued in monetary terms? | Yes | Costs were calculated based on the most recent costing manual with reference year and inflation correction detailed in *Supplementary File 1.* |
|  | b) If not, is a justification provided for not valuing certain components of resource use? | NA |  |
| Time horizon | 10) a) Is the chosen time horizon specified? | Yes | See *Study design and setting, page 4.*  All data in this study are first-visit data. Costs are enquired regarding the past three months, which are extrapolated to 1 year. Data was gathered over 7,5 years, without repeated measures. |
|  | b) If so, is the chosen time horizon justified? | Yes | See end of *Study design and setting, page 4*. |
| Discounting | 11) a) Are future costs discounted? | NA | Not applicable, as the time horizon was not beyond 1 year. |
|  | b) If so, is a justification provided for the discount rate? | NA |  |
| Sensitivity | 12) a) Are all variables whose values are uncertain subjected to sensitivity analysis? | No | All variables were included in a complete case analysis detailed in *Supplementary File 2* and subgroup analysis of *Table 7*. |
|  | b) If so, is a justification provided for which variables are subjected to sensitivity analysis? | NA | As it involved a complete case analysis, all variables were subjected to it that were in the original analysis. |
|  | c) Are analyses done on relevant subgroups? | Yes | See *Table 7, page 13-14.* |
| Results and reporting |  |  |  |
| Cost sectors | 13) Are the study results presented transparently by cost category/sector? | Yes | The results of both cost items were presented separately and combined. *See Results: Cost-of-illness, page 9.* |
| Generalizability | 14) Do the authors discuss the generalizability of study results (e.g., comparing the results to other patient/client groups or/in other settings)? | Partially | See *Discussion: Strengths and limitations, page 18.* Generalizability was discussed but variation of groups was not reported upon. |
| Limitations | 15) Do the authors discuss important limitations? | Yes | See *Discussion: Strengths and limitations, page 18.* |
| Ethical and  distributional  issues | 16) a) Do the authors discuss ethical issues? | NA | Data was fully anonymous, approved by the ethical committee, and declared non-WMO. |
|  | b) Do the authors discuss distributional issues? | Yes | See *Table 7*, *page 13-14*, *bootstrapped subgroup analyses of cost-of-illness, page 15,* and *Discussion, page 16.* |
| Conflict of interest | 17) Do the authors report any potential conflicts of interest? | None to declare | See *Conflict of interest, page 19.* |
| * Suggested answer categories: Yes, No, Partially, Not Applicable (NA), and Unclear | | | |

*From:*

Schnitzler, L., Roberts, T. E., Jackson, L. J., Paulus, A. T., & Evers, S. M. (2023). A consensus-based checklist

for the critical appraisal of cost-of-illness (COI) studies. *International Journal of Technology Assessment in Health Care*, *39*(1), e34. <https://doi.org/10.1017/S0266462323000193>
